# Supplementary material for: Causal Relationship between Aging and Anorexia Nervosa: A White-Matter-Microstructure-Mediated Mendelian Randomization Analysis
Source: Biomedicines. 2024 Aug 16;12(8):1874. doi: 10.3390/biomedicines12081874 (PMC11351342; doi:10.3390/biomedicines12081874)
Supplement: Supplementary file 1 [file biomedicines-12-01874-s001.zip › Supplementary Figure/Figure S8/Leaveoneout plot of MR analysis for TL to ubm-b-2005.pdf]

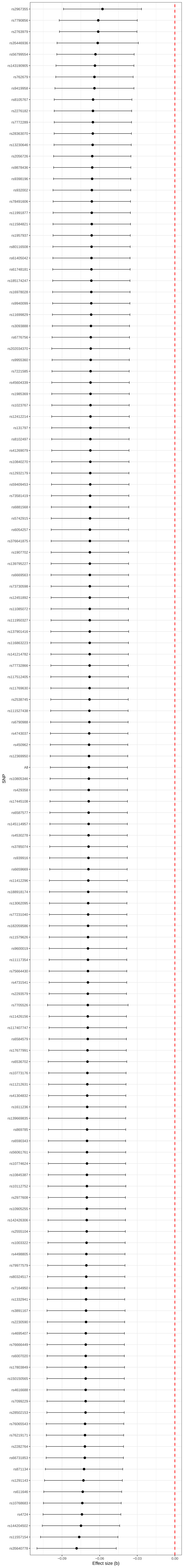
SNP
rs2967355
rs7790856
rs2763979
rs35446936
rs56799554
rs143190905
rs762679
rs9419958
rs8105767
rs2276182
rs7772289
rs28363070
rs13230646
rs2056726
rs9878436
rs9398196
rs932002
rs78491606
rs11991877
rs11584821
rs1957937
rs80116508
rs61405042
rs61748181
rs185174247
rs16978028
rs9940099
rs11699829
rs3093888
rs6776756
rs202034370
rs9955360
rs7221585
rs45604339
rs1985369
rs1023767
rs12412214
rs131797
rs8102497
rs41269079
rs10840270
rs12932179
rs59409453
rs73581419
rs6881568
rs5742915
rs6054257
rs376641875
rs1907702
rs139795227
rs6669563
rs73730598
rs12451892
rs11085072
rs111950327
rs137901416
rs116863223
rs141214782
rs77732866
rs117512405
rs11769630
rs2538745
rs111527438
rs6790988
rs4743037
rs450962
rs12369950
All
rs10805346
rs429358
rs17445108
rs6587577
rs145114957
rs4530278
rs3785074
rs939916
rs6659669
rs11412296
rs188918174
rs13062095
rs77231040
rs182059566
rs11579626
rs9600019
rs11117354
rs75664430
rs4731541
rs2293579
rs7705526
rs11426156
rs117407747
rs6584579
rs17677991
rs6536702
rs10773176
rs11212631
rs41304832
rs1611236
rs139669835
rs869785
rs6590343
rs56061761
rs10774624
rs10845387
rs10112752
rs2977608
rs10905255
rs142426306
rs2555104
rs1003322
rs4498805
rs79977579
rs80324517
rs7164950
rs1332941
rs3891167
rs2230590
rs4695407
rs76666449
rs6007020
rs17803849
rs150150565
rs4616688
rs7099229
rs28502153
rs76065543
rs76219171
rs2282764
rs66731853
rs871134
rs1291143
rs611646
rs10768683
rs4724
rs144204502
rs11557154
rs35640778
-0.09
-0.06
-0.03
0.00
Effect size (b)
